# Effects of Inducing Gamma Oscillations in Hippocampal Subregions DG, CA3, and CA1 on the Potential Alleviation of Alzheimer’s Disease-Related Pathology: Computer Modeling and Simulations

**DOI:** 10.3390/e21060587

**Published:** 2019-06-13

**Authors:** Dariusz Świetlik, Jacek Białowąs, Janusz Moryś, Ilona Klejbor, Aida Kusiak

**Affiliations:** 1 Intrafaculty College of Medical Informatics and Biostatistics, Medical University of Gdańsk, 1 Debinki St., 80-211 Gdańsk, Poland; 2 Department of Anatomy and Neurobiology, Medical University of Gdańsk, 1 Debinki St., 80-211 Gdańsk, Poland; 3 Department of Periodontology and Oral Mucosa Diseases, Medical University of Gdańsk, 18 E. Orzeszkowej St., 80-204 Gdańsk, Poland

**Keywords:** entropy, neural networks, Alzheimer’s disease, hippocampus, long-term potentiation (LTP), gamma oscillations, computer simulation

## Abstract

The aim of this study was to evaluate the possibility of the gamma oscillation function (40–130 Hz) to reduce Alzheimer’s disease related pathology in a computer model of the hippocampal network dentate gyrus, CA3, and CA1 (DG-CA3-CA1) regions. **Methods**: Computer simulations were made for a pathological model in which Alzheimer’s disease was simulated by synaptic degradation in the hippocampus. Pathology modeling was based on sequentially turning off the connections with entorhinal cortex layer 2 (EC2) and the dentate gyrus on CA3 pyramidal neurons. Gamma induction modeling consisted of simulating the oscillation provided by the septo-hippocampal pathway with band frequencies from 40–130 Hz. Pathological models with and without gamma induction were compared with a control. **Results**: In the hippocampal regions of DG, CA3, and CA1, and jointly DG-CA3-CA1 and CA3-CA1, gamma induction resulted in a statistically significant improvement in terms of increased numbers of spikes, spikes per burst, and burst duration as compared with the model simulating Alzheimer’s disease (AD). The positive maximal Lyapunov exponent was negative in both the control model and the one with gamma induction as opposed to the pathological model where it was positive within the DG-CA3-CA1 region. Gamma induction resulted in decreased transfer entropy in accordance with the information flow in DG → CA3 and CA3 → CA1. **Conclusions**: The results of simulation studies show that inducing gamma oscillations in the hippocampus may reduce Alzheimer’s disease related pathology. Pathologically higher transfer entropy values after gamma induction returned to values comparable to the control model.

## 1. Introduction

Gamma oscillations in the hippocampus are nested in slower theta oscillations and are associated with cognitive function, working memory, and learning, among other functions [1,2,3,4,5,6,7,8,9,10,11,12,13]. The results of several studies confirm that there are two independent generators of gamma waves in the hippocampus, which are located in the dentate gyrus (DG) and CA3-CA1 regions [1]. In the hippocampus, the amplitude and frequency of gamma waves may vary from region to region and function independently in DG, CA3, and CA1 regions [14,15,16]. 

Studies in 5XFAD/PV-Cre mice showed that optogenetic stimulation at a frequency of 40 Hz decreased the levels of amyloid-β (Aβ) peptide and induced changes in microglia morphology by increasing Aβ in these glial cells [13,14,15,16]. In another study, Roy et al. showed that optogenetic stimulation with high gamma frequency (100 Hz) reduced memory impairment in APP/PSEN1 mice [17]. In turn, Xia et al. (2017) performed electrical stimulation of entorhinal cortex layer (EC) cells at a frequency of 130 Hz in a mouse TgCRND8 model (mice genetically engineered to model Alzheimer’s disease (AD)). The frequency of this stimulation was above the range of biological gamma oscillation (60–100 Hz), and they found that EC stimulation resulted in blocking accumulation of consecutive Aβ and reduction of cognitive deficits [18]. The results of these studies indicate the role of gamma oscillations in reducing not only AD-related cognitive impairment but also AD-related pathology, and they were inspired by studies by Laxton et al. and Lozano et al. [19,20]. Disturbed gamma waves associated with a decline in cognitive function have been observed in neurodegenerative diseases such as AD [21,22,23]. In experiments simulating memory processes in the Alzheimer’s disease brain, we distinguish two models of neural networks: biophysical- and connection-oriented [24,25,26,27]. Models of neural systems are used in simulations of memory dysfunctions (for example AD) where gamma oscillation is required as well as various types of neurons. It was proven that both of those models clearly presented some universal behaviors depending on the manipulations of network factors. Application of gamma and theta oscillations with different numbers and types of neurons were used in simulations of memory disorders in Alzheimer’s disease [28,29,30].

Methods describing the complexity of biological systems such as the hippocampus use positive Lyapunov exponents, correlative dimensions, Shannon entropy, entropy transfer, and mutual information [31,32,33,34], among others. In many studies of patients with AD, the usefulness of entropy in the analysis of electroencephalography (EEG) signals has been demonstrated [35,36,37,38,39]. Studies have been done on experimental treatment strategies in humans with the application of deep brain stimulation to the fornix–fimbria system, but despite Hz stimulation values they do have not any similarity with gamma frequencies from the EC to the dentate gyrus. In the fornix there are reciprocal connection loops between the septum and the hippocampus with various neurotransmitters. We modeled in our experiments disinhibitory inputs from medial septum–diagonal band gamma-aminobutyric acid (GABA)ergic cells on hippocampal interneurons provided at a theta frequency of 8 Hz [20,40].

Because there is a lack of studies on the influence of gamma stimulation in Alzheimer’s disease, our goal was to determine its effect on the pathology of memory processes in the hippocampus in a computer model. An understanding of these mechanisms can have very important implications for possible future therapeutic interventions in patients with neurodegenerative diseases such as Alzheimer’s.

The aim of this study was to examine the hypothesis that inducing the gamma oscillation function (40–130 Hz) could potentially reduce the symptoms of synaptic breakdown in AD using computational methods describing the complexity of systems.

## 2. Materials and Methods 

### 2.1. Study Design

**Figure 1:**
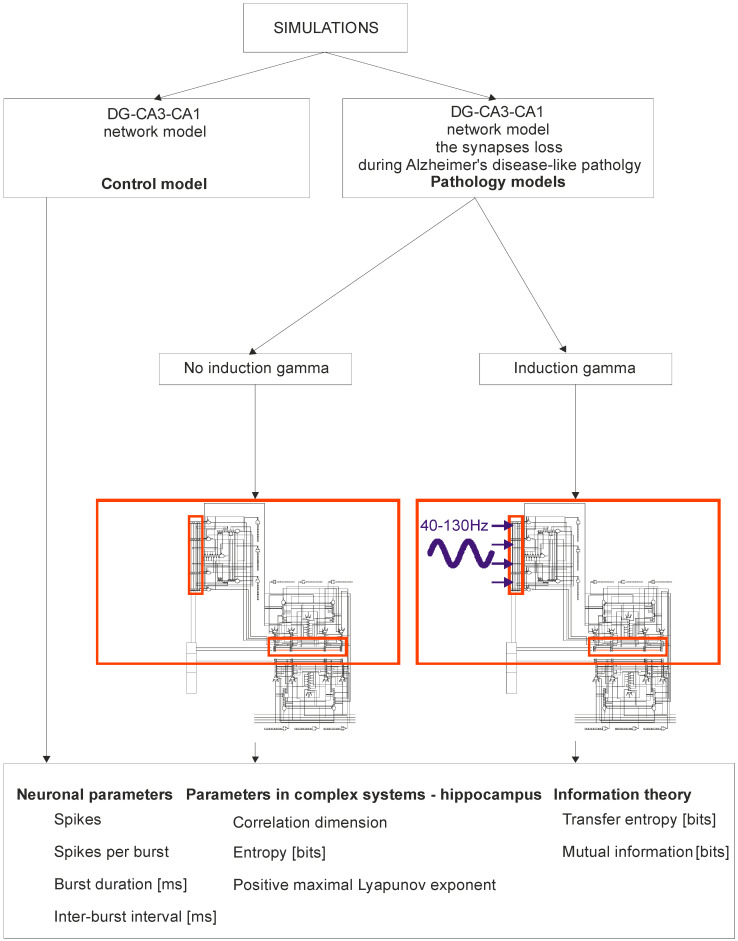
Simulation Diagram of the dentate gyrus (DG)-CA3-CA1 hippocampal network (control model vs. pathology model with and without gamma induction (40–130 Hz); red rectangle represents thumbnail network).

**Figure 2:**
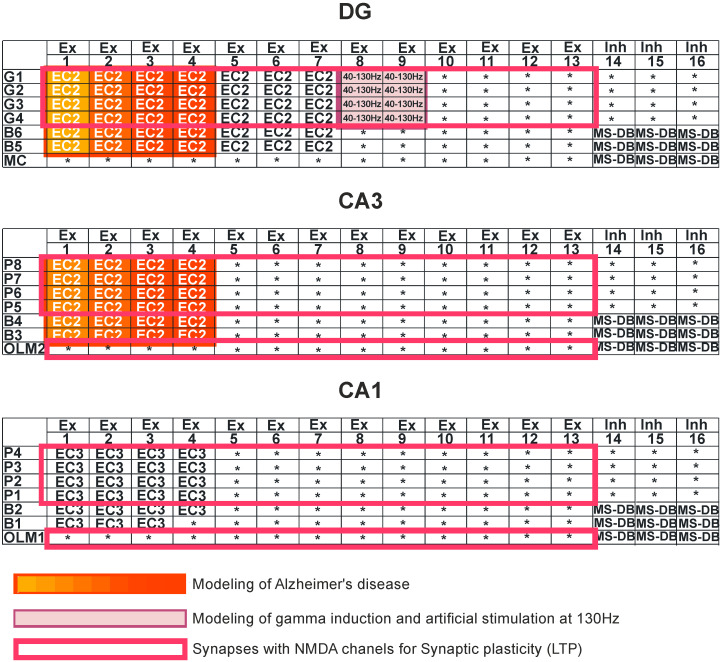
Simulation study. Dentate gyrus (DG): granule cells (G1–G4), basket cells (B5–B6), and mossy cell (MC). CA3 region: pyramidal cells (P5–P8), basket cells (B3–B4), and oriens-lacunosum/moleculare (O-LM2). CA1 region: pyramidal cells (P1–P4), basket cells (B1–B2), and oriens-lacunosum/moleculare (O-LM1). Ex—excitatory inputs, Inh—inhibitory inputs, and MS-DB—medial septum-diagonal band region. Modeling of Alzheimer’s disease was based on sequentially turning off connections from EC2 on granule cells of the dentate gyrus and pyramidal neurons of the CA3 region as well as on inhibitory interneurons (interrupted connections marked in red in DG and CA3 regions). The deletions were made only in DG-CA3 microcircuits. Modeling of gamma induction consisted of simulating oscillation of the perforant pathway from entorhinal cortex layer 2 (EC2) to the dentate gyrus and CA3 region, with band frequencies from 40–130 Hz (marked in purple).

**Figure 3:**
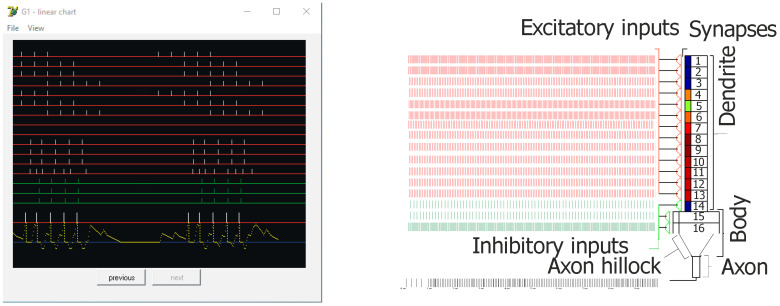
Screen capture from the simulation showing an example of inputs and outputs of the principal cell (Granule G1—control) on left. On the right, a general diagram of the used neuron model. Red lines (Ex 1–13) represent excitatory inputs, green lines (Inh 14–16) represent inhibitory inputs, and bars above the red threshold line (left–bottom) mean output spikes (action potentials—two bursts of five spikes). Connections from EC2 to granule cells are shown at Ex 1–9. For Ex 1–7, there are bursts of five action potentials (100 Hz) with inter-burst theta frequency at 8 Hz, shifted in phase between particular lines. On lines Ex 8 and 9 there were no spikes (silent synapses). For experiments, gamma oscillations were added at 40, 100, or 130 Hz. On the pyramidal cell model configuration on the right, small rectangles below synapses mean postsynaptic thickenings. Their color changes show the actual weight of particular excitatory synapses, which could be observed on the line for each cell during simulation.

Figure 1 shows a detailed diagram of a simulation of the DG-CA3-CA1 hippocampus network. Computer simulations were performed for the control model and for pathological models; two models were for the induction of gamma oscillations, and one model was for no induction. In brief, modeling of Alzheimer’s disease was based on sequentially turning off connections from EC2 on granule cells of the dentate gyrus and pyramidal neurons of the CA3 region as well as on inhibitory interneurons (interrupted connections marked in Figure 2). There were only computer simulations in agreement with previous statements about staging of Alzheimer’s pathology in humans, especially “preclinical” stages “0” and “1” with very mild memory impairment, but degeneration of some stellate cells in EC2 already existed in practically all humans above 60 years of age [41,42] (Figure 2). Modeling of gamma induction consisted of simulating oscillation of the perforant pathway from EC2 to the dentate gyrus and CA3 region with band frequencies from 40–100 Hz and with 130 Hz after [18] (Figure 2 and Figure 3). Our simulation studies allowed comparisons of neuronal parameters, highlighting the complexity of systems such as the hippocampus, and information theories.

### 2.2. Dentate Gyrus (DG)-CA3-CA1 Model

**Figure 4:**
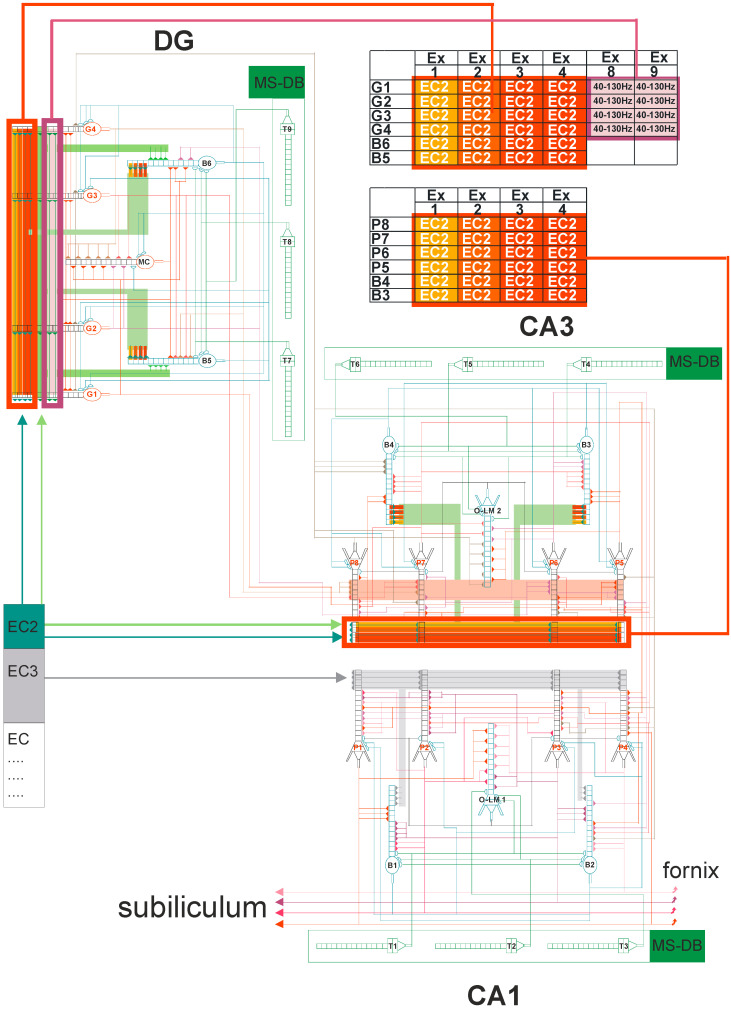
DG-CA3-CA1 hippocampal formation microcircuit, with the dentate gyrus (DG) region on the left and CA3 on the right. Major cell types and their connectivity: granule (G1–G4), pyramidal (P5–P8), basket (B3–B6), oriens-lacunosum/moleculare (O-LM) (2) cell, and mossy cell (MC). T4–T9 represent gamma-aminobutyric acid (GABA)ergic cells in the medial septum-diagonal band (MS-DB) region. Pyramidal, granule, basket, and O-LM cells consist of 16 compartments with 13 excitatory and 3 inhibitory synapses. Simplified demo version of FC-neuron model available on ModelDB (https://senselab.med.yale.edu/modeldb) or (https://medinf.gumed.edu.pl/383.html). With simultaneous (only on granule, pyramidal, and O-LM cells) action potential on excitatory input and opened N-methyl-D-aspartate (NMDA) channels, since there is enough depolarization of the postsynaptic region, long-term potentiation (LTP) induction occurs, and the weight of this synapse is increased.

The neural network scheme associated with DG-CA3-CA1 hippocampal subregions uses mathematical formulas based on previous studies [43,44,45,46,47,48,49]. The detailed hippocampal network organization is presented in Figure 4 from a recently published paper in *Entropy* [49]. In brief, our neural network model consisted of 21 cells, with 4 granule cells, 2 inhibitory interneurons (2 basket cells), and 1 mossy cell in the DG region, and 4 pyramidal cells, 3 inhibitory interneurons (2 basket cells, and an oriens-lacunosum/moleculare (O-LM) cell in the CA3 and CA1 regions. Figure 4 shows an abbreviated diagram of DG and CA3 connections needed for a detailed explanation of the simulation experiments as shown in Figure 2. The morphology of nerve cells was based on simplification, which included the cell body, part of the axon, and dendrites with the properties used in the experiments described in the literature [50,51,52,53,54,55].

### 2.3. Synaptic Properties

In our DG-CA3-CA1 neural network model, pyramidal, basket, and O-LM cells were built with 16 compartments. In addition, dendrite had both excitatory and inhibitory synapses. We used computational formality, which characterized the α-amino-3-hydroxy-5-methyl-4-isoxazolepropionic acid receptor (AMPA), N-methyl-D-aspartate receptor (NMDA), and receptors that responded to GABA from previous works [46,47,48,49].

### 2.4. Correlation Dimension, Shannon Entropy, Positive Maximal Lyapunov Exponent, Mutual Information, and Transfer Entropy

Nonlinear analysis of the results of the control model simulation of pathologies allowed for the reconstruction of the phase space as a method to describe the complexity of the dynamic system [56]. Reconstruction of the attractor used the time delay method [57,58]. In contrast, the method of false nearest neighbors selected a minimum dimension of deposition of a one-dimensional time series of simulation results of neural networks [59]. The final stage was the calculation of correlation dimension, Shannon entropy, and the positive maximal Lyapunov exponent with the method of recurrence quantification analysis proposed by Webber and Zbilut [59]. The Shannon entropy of time series of simulations was based on [60].

In terms of information theory, mutual information was designated as an alternative to correlation analysis [61,62,63]. However, because mutual information measures how much information we can have about signal A knowing B, but does not provide knowledge about the dynamics and direction of flow, the method of entropy transfer was used [33,34].

### 2.5. Statistical Methods and Software

Statistical analysis was performed using TIBCO Software Inc. (2017), Statistica (data analysis software system), version 13, (Palo alto, CA, USA, 2017, http://statistica.io). The significance of difference between more than two groups was assessed with either Fisher or Kruskal–Wallis tests. For statistically significant differences between two groups, post hoc tests were used. Chi-squared tests for independence were used for qualitative variables. In order to determine dependence, strength, and direction between variables, a correlation analysis was used by determining the Pearson or Spearman’ correlation coefficients. All calculations used a statistical significance level of α = 0.05. Parameter calculations for complex systems and information theory were made in Neuroscience Information Theory Toolbox software (Version 2 (2017)) [33].

## 3. Results

### 3.1. Neuronal Parameters

In simulations comparing the control model with pathological models with and without induction of gamma oscillation, the following parameters were used: number of spikes, spikes per burst, burst duration, and inter-burst interval. These parameters were compared for both the DG-CA3-CA1 and CA3-CA1 hippocampal regions as well as for the DG, CA3, and CA1 regions separately.

#### 3.1.1. DG-CA3-CA1

**Figure 5:**
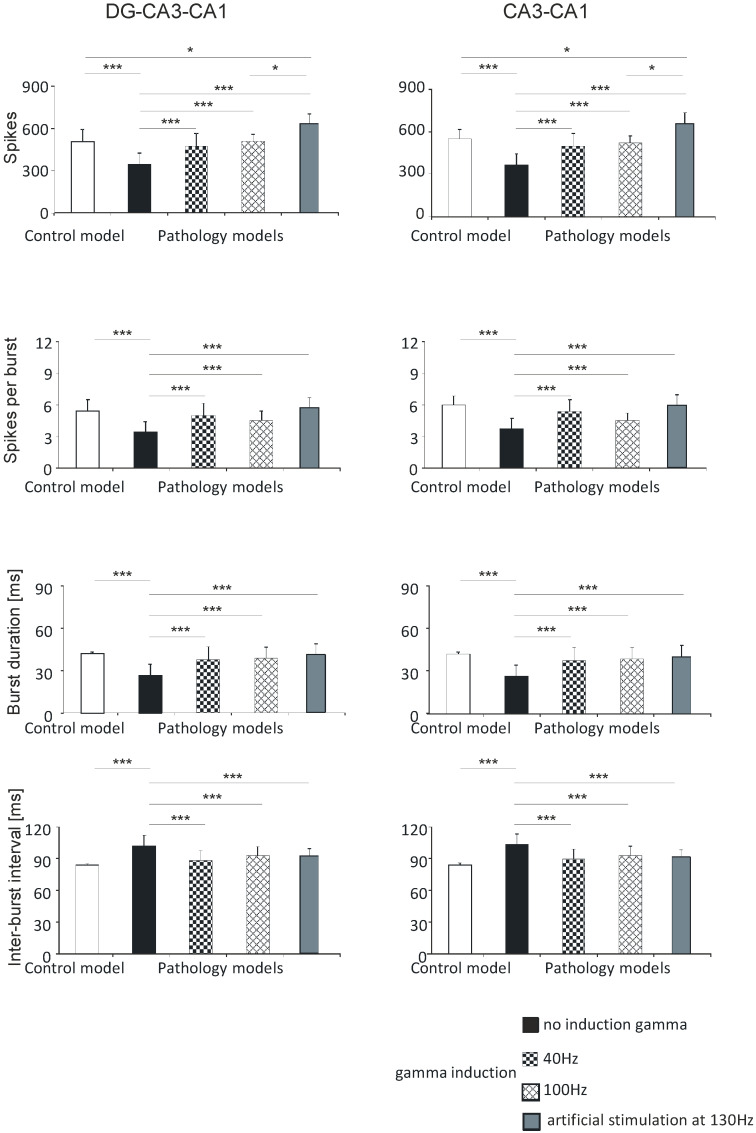
Analysis of simulations of DG-CA3-CA1 (left) and CA3-CA1 (right). Number of spikes, spikes per burst, burst duration, and inter-burst interval for pyramidal cell compared with control model and pathology model, with and without induction (40–130 Hz) (* *p* < 0.05; ** *p* < 0.01; *** *p* < 0.001; ns, not statistically significant).

The mean values for number of spikes obtained in computer simulations were 505.0 (standard deviation (SD) 86.4) in the control model, 342.2 (81.3) in the pathological model without gamma induction, 469.4 (92.7) with gamma induction (40 Hz), 513.4 (46.5) with gamma induction (100 Hz), and 630.1 (71.0) with artificial stimulation (130 Hz). The pathological model without gamma induction showed a significantly lower number of spikes compared to the control (*p* < 0.001) and the pathological models with induction (40–130 Hz) (*p* < 0.001). A comparison of the pathological models with gamma induction (40–100 Hz) relative to the control did not show a statistically significant difference in number of spikes. The pathological model with artificial induction at 130 Hz showed a significantly higher number of spikes compared to the control (*p* < 0.05) and the pathological model with induction (40 Hz) (*p* < 0.001) (Figure 5).

Similar associations were obtained for spikes per burst. In the control model the value was 5.4 (1.1); in the pathological model without gamma induction it was 3.4 (1.0), with gamma induction (40 Hz) it was 5.0 (1.2), with gamma induction (100 Hz) it was 4.5 (0.9), and with induction (130 Hz) it was 5.7 (1.0). The pathological model without gamma induction showed significantly fewer spikes per burst relative to the control (*p* < 0.001) and the pathological models with induction (40–130 Hz) (*p* < 0.001). A comparison of the pathological models with induction with the control did not show a statistically significant difference in spikes per burst (Figure 5).

Calculations comparing burst duration showed that the control was 41.9 (1.3); the pathological model without gamma induction was 26.6 (7.8), with gamma induction (40 Hz) it was 37.8 (9.2), with gamma induction (100 Hz) it was 39.4 (7.6), and with induction (130 Hz) it was 40.9 (7.8). The pathological model without gamma induction showed a significantly lower burst duration relative to the control (*p* < 0.001) and the pathological models with induction (*p* < 0.001). A comparison of the pathological models with induction (40–130 Hz) with the control did not show a statistically significant difference in burst duration (Figure 5).

According to predictions, the inter-burst interval parameter for the control was 84.1 (1.3); in the pathological model without gamma induction it was 102.1 (10.3), with gamma induction (40 Hz) it was 88.8 (9.0), with gamma induction (100 Hz) it 93.4 (8.7), and with artificial induction (130 Hz) it was 91.7 (7.2). The pathological model without gamma induction showed a significantly greater inter-burst interval relative to the control (*p* < 0.001) and the pathological models with induction (*p* < 0.001) (Figure 3). A comparison of the pathological models with induction with the control did not show a statistically significant difference in inter-burst intervals (Figure 5).

There were no statistically significant differences between number of spikes per burst, burst duration, inter-burst interval, and hippocampal models with induction (40–130 Hz) in the DG-CA3-CA1 region.

#### 3.1.2. CA3-CA1

In the CA3-CA1 hippocampal region, the number of spikes was 550.6 (67.3) in the control model, 364.4 (79.4) in the pathological model without gamma induction, 499.7 (88.7) with gamma induction (40 Hz), 523.4 (76.7) with gamma induction (100 Hz), and 658.0 (77.1) with artificial induction (130 Hz). The number of spikes per burst in the control model was 6.0 (0.9); in the pathological model without gamma induction it was 3.7 (1.0), with gamma induction (40 Hz) it was 5.4 (1.1), with gamma induction (100 Hz) it was 4.5 (0.7), and with induction (130 Hz) it was 5.9 (1.0). The burst duration in the control was 42.1 (1.6); in the pathological model without gamma induction it was 26.3 (8.0), with gamma induction (40 Hz) it was 37.5 (9.3), with gamma induction (100 Hz) it was 39.6 (7.9), and with induction (130 Hz) it was 40.2 (8.1). The inter-burst interval parameter in the control was 84.3 (1.6); in the pathological model without gamma induction it was 103.2 (10.2), with gamma induction (40 Hz) it was 89.6 (9.4), with gamma induction (100 Hz) it was 93.5 (8.7), and with induction (130 Hz) it was 91.3 (6.5) (Figure 5). The results of the statistical tests were the same as those for the corresponding parameters in the DG-CA3-CA1 region (Section 3.1.1, DG-CA3-CA1).

There were no statistically significant differences between number of spikes per burst, burst duration, inter-burst interval, and hippocampal models with induction (40–130 Hz) in the CA3-CA1 region.

#### 3.1.3. DG, CA3, and CA1

**Figure 6:**
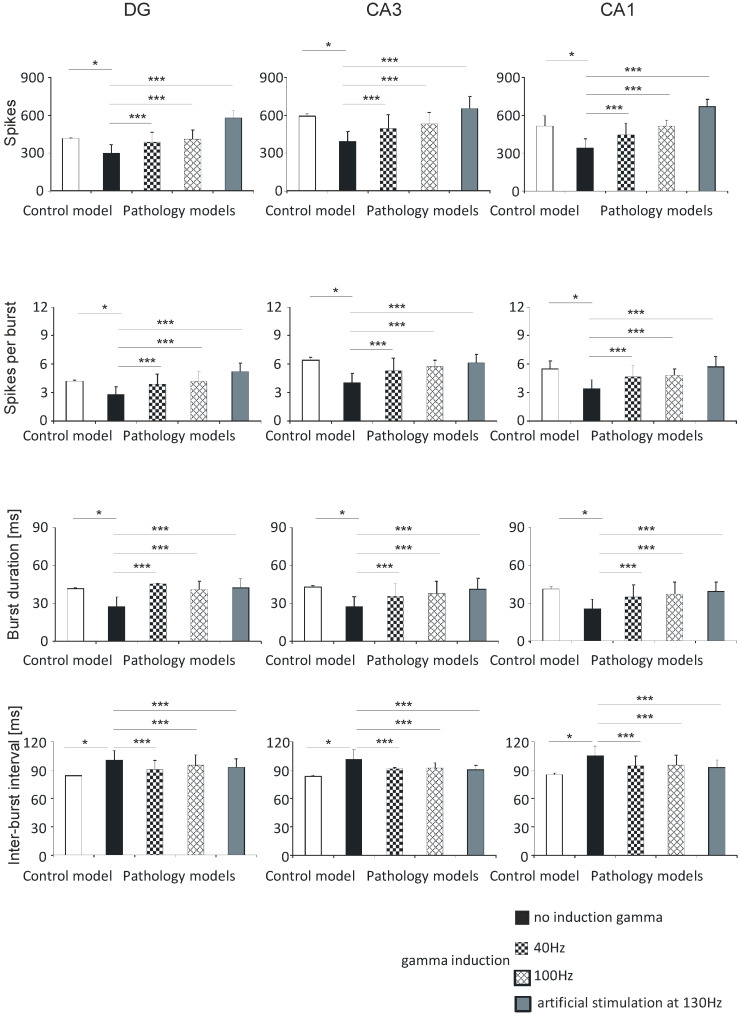
Analysis of simulations DG, CA3, and CA1. Number of spikes, spikes per burst, burst duration, and inter-burst interval for pyramidal cells comparing control model and pathology model with and without induction (40–130 Hz) (* *p* < 0.05; ** *p* < 0.01; *** *p* < 0.001; ns, not statistically significant).

In the DG hippocampal region, the number of spikes was 413.0 (5.0) in the control model, 297.8 (67.2) in the pathological model without gamma induction, 384.4 (79.6) and 409.6 (69.3) with gamma induction (40 and 100 Hz), and 574.3 (58.9) with artificial stimulation at 130 Hz. The number of spikes in the CA3 region was 587.8 (20.1) in the control model, 388.0 (80.2) in the pathological model without gamma induction, 492.2 (108.0) and 526.5 (92.8) with gamma induction (40 and 100 Hz), and 649.7 (96.3) with artificial stimulation at 130 Hz. The corresponding values in the CA1 region were 513.5 (80.5) in the control, 340.8 (73.6) in the pathological model without gamma induction, 443.1 (94.0) and 513.6 (76.5) with gamma induction (40 and 100 Hz), and 666.3 (57.8) with artificial stimulation at 130 Hz (Figure 6).

The number of spikes per burst in the DG region was 4.2 (0.1) in the control model, 2.8 (0.8) in the pathological model without gamma induction, 3.9 (1.0) and 4.2 (0.7) with gamma induction (40 and 100 Hz), and 5.2 (0.9) with artificial stimulation at 130 Hz. The values in the CA3 region were 6.4 (0.3) in the control, 4.0 (1.0) in the pathological model without induction, 5.3 (1.3) and 5.7 (0.7) with induction (40 and 100 Hz), and 6.1 (0.9) with artificial stimulation at 130 Hz. The values in the CA1 region were 5.5 (1.0) in the control, 3.4 (0.9) and 4.7 (1.2) in the pathological models (40 and 100 Hz), and 5.7 (1.1) with artificial stimulation at 130 Hz (Figure 6).

The burst duration values of the DG region were 41.6 (0.5) in the control model, 27.3 (7.8) in the pathological model without gamma induction, 45.3 (0.5) and 40.7 (6.8) with gamma induction (40 and 100 Hz), and 42.3 (7.3) with artificial stimulation at 130 Hz. The values in the CA3 region were 43.0 (1.1) in the control, 27.2 (8.2) in the pathological model without induction, 35.4 (10.0) and 37.9 (9.6) with gamma induction (40 and 100 Hz), and 41.2 (8.7) with artificial stimulation at 130 Hz. In the CA1 region, the values were 41.2 (1.7) in the control, 25.3 (7.8) in the pathological model without induction, 34.5 (9.9), and 37.1 (9.4) in the pathological models (40 and 100 Hz), and 39.2 (7.4) with artificial stimulation at 130 Hz (Figure 6).

The inter-burst interval values of the DG region were 83.7 (0.5) in the control model, 99.9 (10.3) in the pathological model without gamma induction, 90.3 (9.6) and 94.4 (11.2) with gamma induction (40 and 100 Hz), and 92.6 (8.7) with artificial stimulation at 130 Hz. In the CA3 region, they were 83.4 (1.1) in the control, 101.2 (10.3) in the pathological model without induction, 91.7 (10.7) and 92.4 (5.2) in the pathological models with induction (40 and 100 Hz), and 90.2 (4.6) with artificial stimulation at 130 Hz. In the CA1 region the values obtained were 85.2 (1.7), 105.2 (10.0), 94.1 (11.0) and 95.3 (10.6), and 92.3 (8.4), respectively (Figure 6).

The results of the statistical tests were the same as those for the corresponding parameters in the DG-CA3-CA1 region (Section 3.1.1) and CA3-CA1 region (Section 3.1.2).

There were no statistically significant differences between number of spikes per burst, burst duration, inter-burst interval, and hippocampal models with induction (40–100 Hz) in the DG, CA3, and CA1 regions.

### 3.2. Parameters in the Complex System: Hippocampus

The parameters of correlation dimension, Shannon entropy, and positive maximal Lyapunov exponent were compared between the control model and the pathological models with and without gamma induction.

#### 3.2.1. DG-CA3-CA1

In the DG-CA3-CA1 hippocampal region, the correlation dimension was 6.0 (1.5) in the control model, 5.3 (2.7) in the pathological model without gamma induction, 5.4 (2.3) and 6.1 (2.4) with gamma induction (40 and 100 Hz), and 5.9 (1.9) with artificial stimulation at 130 Hz. There were no statistically significant differences in the correlation dimension of the models (*p* > 0.05).

Shannon entropy was 1.9 (1.3) in the control model, 2.2 (1.1) in the pathological model without gamma induction, 0.9 (0.3) and 0.9 (0.4) with gamma induction (40 and 100 Hz), and 1.0 (0.3) with artificial stimulation at 130 Hz. In the pathological model with induction, a statistically significant decrease in entropy was seen relative to the model without induction (*p* < 0.001) and to control (*p* < 0.01).

**Figure 7:**
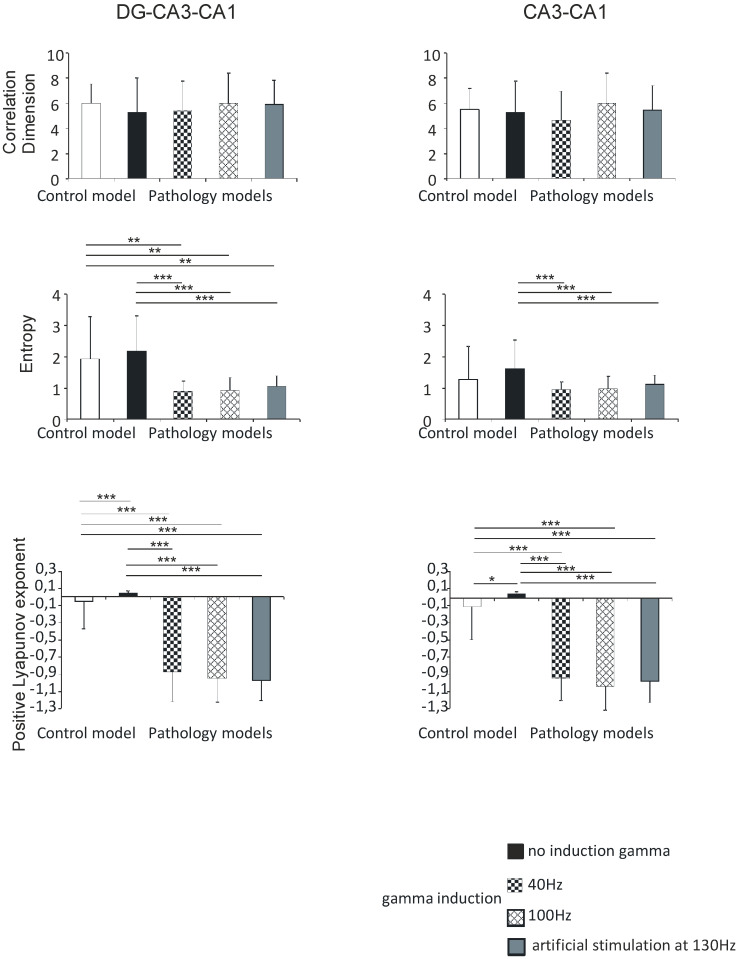
Nonlinear analysis of simulations of DG-CA3-CA1 region (left) and CA3-CA1 (right). Comparison of control model and pathological model with and without induction (40–130 Hz); correlation dimension, entropy, and positive Lyapunov exponent for pyramidal cells (* *p* < 0.05, ** *p* < 0.01, and *** *p* < 0.001).

Positive Lyapunov exponents were −0.1 (0.3) in the control model, 0.1 (0.1) in the pathological model without gamma induction, −0.9 (0.3) and −1.0 (0.3) with gamma induction (40 and 100 Hz), and −1.0 (0.2) with artificial stimulation at 130 Hz. In the pathological models with induction, a statistically significant decrease in the positive Lyapunov exponent was seen relative to the model without induction (*p* < 0.001) and the control (*p* < 0.001) (Figure 7).

There were no statistically significant differences between correlation dimension, Shannon entropy, positive Lyapunov exponents, and hippocampal models with gamma induction (40 and 100 Hz) in the DG-CA3-CA1 region.

#### 3.2.2. CA3-CA1

Similar to the DG-CA3-CA1 hippocampal region, there were no statistically significant differences between correlation dimension of models (*p* > 0.05), and the values obtained were 5.5 (1.7) in the control model, 5.3 (2.5) in the pathological model without gamma induction, 4.7 (2.3) and 6.0 (2.4) with gamma induction (40 and 100 Hz), and 5.5 (2.0) with artificial stimulation at 130 Hz.

Shannon entropy in the CA3-CA1 region was 1.3 (1.1) in the control model, 1.6 (0.9) in the pathological model without gamma induction, 0.9 (0.2) and 1.0 (0.4) with gamma induction (40 and 100 Hz), and 1.1 (0.3) with artificial stimulation at 130 Hz. Contrary to the DG-CA3-CA1 region, a statistically significant decrease in entropy in the pathological model with gamma induction compared to the model without induction was seen (*p* < 0.001).

In contrast, positive Lyapunov exponents were −0.1 (0.4) in the control model, 0.1 (0.1) in the pathological model without gamma induction, −0.9 (0.3) and −1.0 (0.3) with gamma induction (40 and 100 Hz), and −1.0 (0.3) with artificial stimulation at 130 Hz. In the pathological models with induction, a statistically significant decrease in the positive Lyapunov exponent relative to the model without induction (*p* < 0.001) and to control (*p* < 0.001) was seen. In addition, the positive Lyapunov exponent of the control model was significantly lower compared to the positive Lyapunov exponent of the pathological model without induction (*p* < 0.05) (Figure 7).

There were no statistically significant differences between correlation dimension, Shannon entropy, positive Lyapunov exponents, and hippocampal models with gamma induction (40–100 Hz) in the CA3-CA1 region.

#### 3.2.3. DG, CA3, and CA1

The correlation dimension values in the DG region were 7.0 (0.1) in the control model, 5.3 (3.3) in the pathological model without gamma induction, 7.0 (1.5) and 6.5 (2.3) with gamma induction (40 and 100 Hz), and 6.8 (1.8) with artificial stimulation at 130 Hz. Correlation dimension was significantly higher in the pathological models with induction compared to the model without induction (*p* < 0.01) and to control (*p* < 0.05). The values in the CA3 region were 5.5 (1.7) in the control and 5.8 (2.5) in the pathological model without induction, 4.6 (1.9) and 6.0 (2.1) in the pathological models with induction (40 and 100 Hz), and 5.8 (2.0) with artificial stimulation at 130 Hz. The values in the CA1 region were 5.5 (1.9), 4.8 (2.4), 4.7 (2.7), and 5.0 (2.3) in the control and pathological models, respectively. The value in the CA3 region was 5.1 (1.9) in the pathological model with artificial stimulation at 130 Hz. In both the CA3 and CA1 regions, there were no statistically significant differences in correlation dimension relative to hippocampus models (*p* > 0.05).

**Figure 8:**
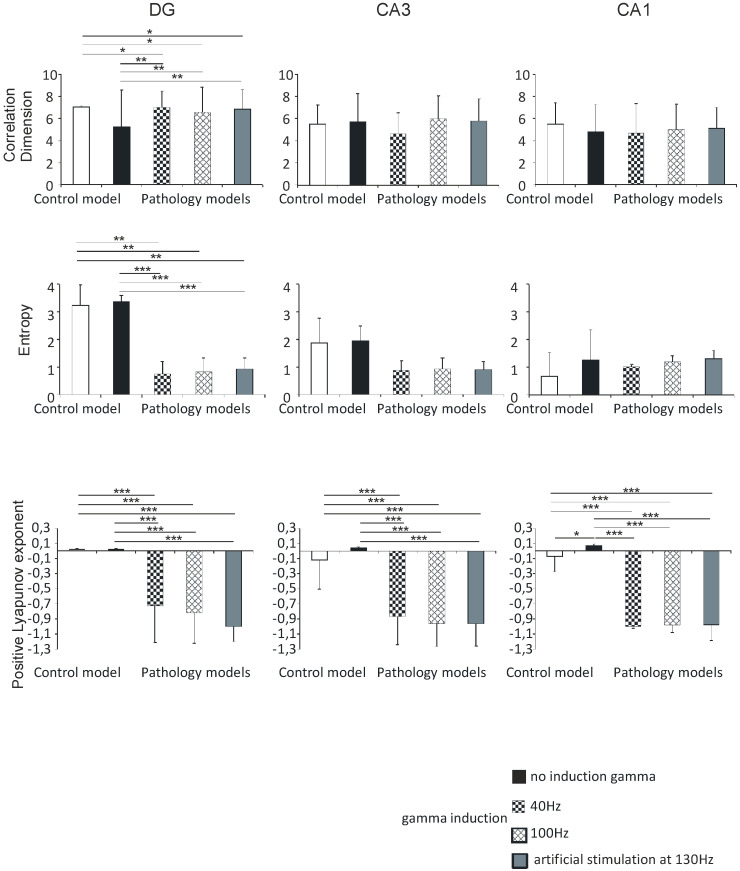
Nonlinear analysis of simulations of DG, CA3, and CA1. Comparison of control model and pathological model with and without induction (40–130 Hz); correlation dimension, entropy, and positive Lyapunov exponent for pyramidal cells (* *p* < 0.05, ** *p* < 0.01, and *** *p* < 0.001).

The Shannon entropy of the DG region was 3.2 (0.7) in the control model, 3.4 (0.2) in the pathological model without gamma induction, 0.8 (0.4) and 0.8 (0.5) with gamma induction (40 and 100 Hz), and 0.9 (0.4) with artificial stimulation at 130 Hz. Shannon entropy was significantly lower in the pathological models with gamma induction than in the model without induction (*p* < 0.001) and the control (*p* < 0.01). In the CA3 region, the values were 1.9 (0.9) in the control, 1.9 (0.5) in the pathological model without induction, 0.9 (0.3) and 0.9 (0.4) in the pathological models with induction (40 and 100 Hz), and 0.9 (0.3) with artificial stimulation at 130 Hz. In the CA1 region, the values were 0.7 (0.9), 1.3 (1.1), 1.0 (0.1), and 1.2 (0.2) in the control and pathological models, respectively. The Shannon entropy of the CA3 region was 1.3 (0.3) in the pathological model with artificial stimulation at 130 Hz. In both CA3 and CA1 regions, there were no statistically significant differences between Shannon entropy and hippocampal models (*p* > 0.05) (Figure 8).

Positive Lyapunov exponents of the DG region were 0.02 (0.01) in the control model, 0.02 (0.01) in the pathological model without gamma induction, and −0.7 (0.5) and −0.8 (0.4) with gamma induction (40 and 100 Hz). In the CA3 region, the values were −0.1 (0.4) in the control, 0.04 (0.01) in the pathological model without induction, −0.9 (0.4) and −1.0 (0.3) with induction (40 and 100 Hz), and −1.0 (0.2) with artificial stimulation at 130 Hz. In the pathological model with induction, we observed a statistically significant decrease in the positive Lyapunov exponent relative to the induction-free model (*p* < 0.001) and control (*p* < 0.001) in both DG and CA3. Positive Lyapunov exponents of the CA1 region were −0.1 (0.4) in the control model, 0.1 (0.03) in the pathological model without gamma induction, −1.0 (0.01) with gamma induction (40 and 100 Hz), and −1.0 (0.3) with artificial stimulation at 130 Hz. In the pathological models with induction, we observed a statistically significant decrease in the positive Lyapunov exponent relative to the model without induction (*p* < 0.001) and control (*p* < 0.001). In addition, the positive Lyapunov exponent of the control model was significantly lower compared to that of the pathological model without induction (*p* < 0.05) (Figure 8).

There were no statistically significant differences between correlation dimension, Shannon entropy, positive Lyapunov exponents, and hippocampal models with gamma induction (40 and 100 Hz) in the DG, CA3, and CA1 regions.

### 3.3. Transfer Entropy and Mutual Information

**Figure 9:**
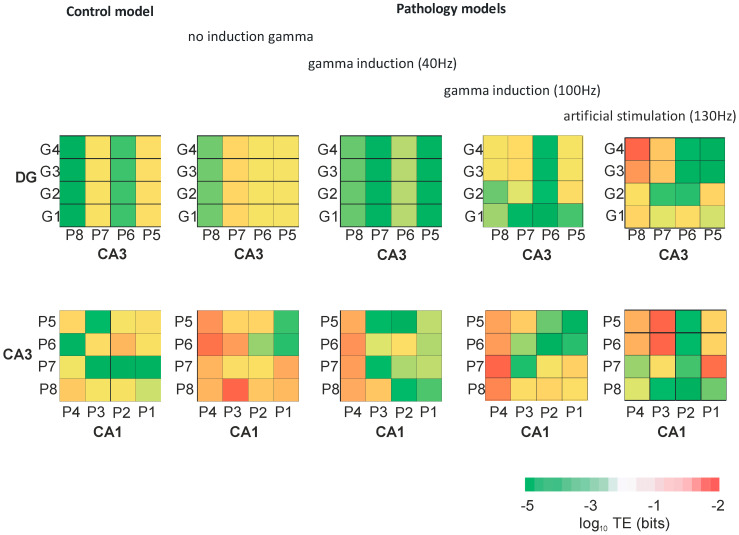
Transfer entropy (TE) between neurons of DG and CA3, and CA3 and CA1 for control and pathological models with and without gamma induction (40–130 Hz) (G1–G4 granule cells of DG, P1–P4 pyramidal cells of CA1, and P5–P8 pyramidal cells of CA3 region).

**Figure 10:**
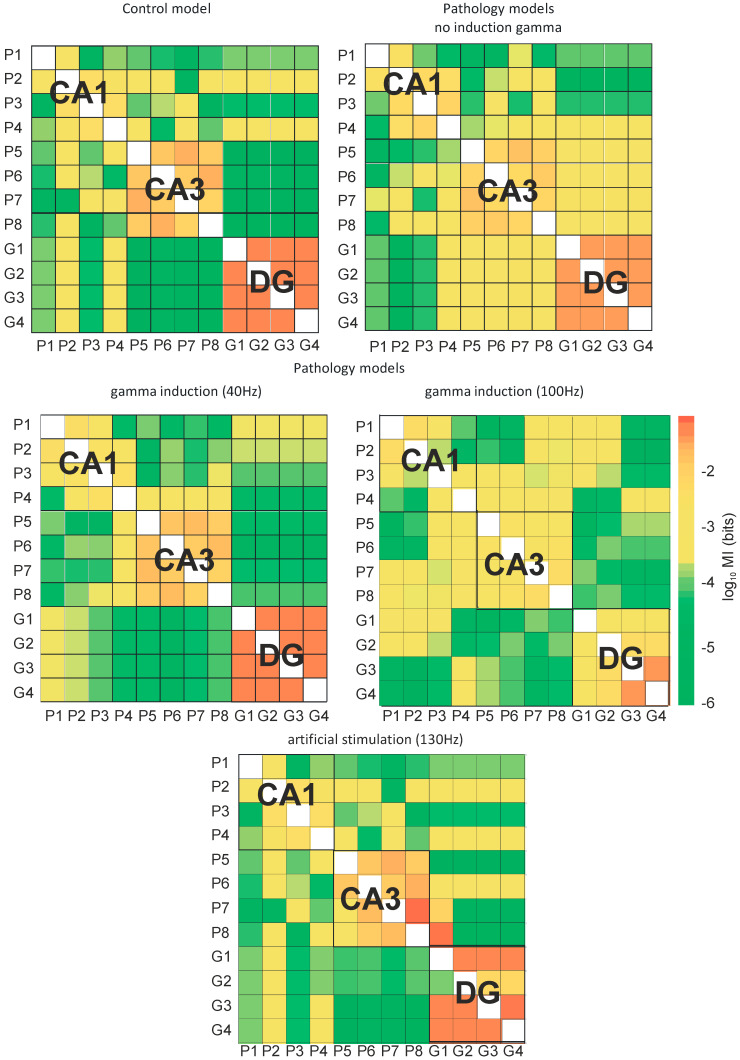
Mutual information (MI) between neurons of hippocampus (DG-CA3-CA1) for control and pathological models with and without gamma induction (40–130 Hz) (G1–G4 granule cells of DG, P1–P4 pyramidal cells of CA1, and P5–P8 pyramidal cells of CA3 region).

Figure 9 presents the transfer entropy of control and pathological models with and without induction (40–130 Hz) between neurons of the DG and CA3 regions as well as CA3 and CA1 regions. Figure 10 provides mutual information between all neurons of the DG, CA3, and CA1 regions in the control and pathological models. In the control and the pathological model with induction, transfer entropy had lower values compared to the pathological model without induction according to the flow of information from DG to CA3. Similarly, there was an increase in transfer entropy in the pathological model without induction relative to the control and the pathological models with induction from CA3 to CA1.

Figure 10 presents the mutual information of the control and pathological models with and without induction (40–130 Hz) between neurons of the DG and CA3 regions as well as CA3 and CA1 regions. Figure 10 presents mutual information between all neurons of the DG, CA3, and CA1 regions of the control and pathological models. In the control and pathological models with gamma induction, transfer entropy had lower values compared to the pathological model without induction according to the flow of information from DG to CA3. Similarly, there was an increase in mutual information in the pathological model without induction relative to the control and the pathological models with induction from CA3 to CA1.

In the control model, mutual information of neurons in the CA1, CA3, and DG regions was very high. However, the relationships between neurons in the DG and CA3 as well as CA3 and CA1 regions were much less. In the pathological model without induction, there was a reduction in the degree of interactions between neurons in the CA3, CA1, and DG regions. However, there was an increase in mutual information of the neurons of DG and CA3. The use of gamma induction (40 Hz) in the pathological model caused mutual information between neurons in particular areas of the hippocampus to have similar values as the control model.

## 4. Discussion

The results of the experiments described in the literature show that gamma oscillations in the hippocampus are very important in cognitive processes [12,64,65]. In our simulation study, we showed that gamma induction in the pathological model of Alzheimer’s disease influenced the improvement of cognitive function in the form of increased number of spikes, spikes per burst, burst duration, and reduced inter-burst interval relative to the model without induction. An interesting confirmation of this improvement was the lack of statistically significant differences in the values of neuronal parameters relative to the control. The results of our simulation experiment can be compared to the results of the study in 5XFAD mice, where the induction of gamma oscillation, using optogenetics, influenced the reduction of Aβ peptides [66]. In addition, reduced gamma oscillation has been observed in many regions of the brain in neurological disorders, including patients with Alzheimer’s disease and many mouse models of AD [13,14,15,16]. 

Hasselmo et al. created a model based on runaway synaptic modification, as a mechanism of degradation of memory in AD [67,68], whereas the models of Menschik and Finkel, inspired by the exponential increase in the strength of synaptic connections, were based on the loss of cholinergic connections in Alzheimer’s disease [24,28,29,30]. Our model of the hippocampal network system was a full connectionist model, which means that each synaptic connection was individually positioned on a particular neuron, and there were various types of neurons and input patterns [46,47,48,49].

A detailed analysis of the nonlinear dynamics of the simulation results of pathological and control models showed that the correlation dimension played a significant role only in the DG region. The complexity of this region was significantly higher in the pathological model with gamma induction relative to the model without induction and the control. It can be concluded that gamma induction, which causes an increase in neuronal parameters, requires more degrees of freedom.

Shannon entropy, considered as the average amount of information, was significantly lower after gamma induction in the pathological model in DG-CA3-CA1, CA3-CA1, and DG regions relative to the model without gamma induction. The calculated Lyapunov exponents showed that the control and the pathological model with gamma induction in DG-CA3-CA1, CA3-CA1, DG, CA3, and CA1 regions had negative values (i.e., they were stable systems). Positive Lyapunov exponents for the pathological model without induction make it an unstable system with chaos. Gamma induction in a pathological model brings the system to a stable point. The usefulness of Shannon entropy and transfer entropy in the analysis of EEG signals in Alzheimer’s disease has been demonstrated. The increased value of Shannon entropy in AD has been shown in previous studies [35,36,37,38].

Information in the hippocampus flows from DG to CA3 and from CA3 to CA1. Although the calculated Shannon information entropy values showed its decline with the direction of information flow for the control and the pathological model without induction, the process of constant dissipation of energy caused its growth for the entire brain. The results of the mutual information analysis showed a very strong linkage between the neurons of DG, CA3, and CA1 areas of the hippocampus, while the interaction of DG with CA3 in the control model was weak. The pathological model without gamma induction showed a weakening of the linkage in the CA3, CA1, and DG areas while enhancing the interaction between DG and CA3. The promising result after the use of gamma induction in the pathological model was a return to conditions close to control.

In our control model simulation experiment, transfer entropy values from DG to CA3 and from CA3 to CA1 fell into the medium range and showed mutual balance. The pathological model without induction indicated an increase in transfer entropy from DG to CA3 with a very strong increase from CA3 to CA1. Gamma (40–100 Hz) induction reduced the symptoms of AD pathology in simulations. The use of gamma induction (40 Hz) in the pathological model caused mutual information between neurons in particular areas of the hippocampus to have similar values as the control model. The use of 130 Hz stimulation of granule cells improved the out-coming firing to values even higher than control simulations without synaptic deletion. It is impossible to compare results of our 10 s model simulation to 1 h entorhinal deep-brain stimulation and to the extensive behavioral experiments during a few months, as seen in Frances Xia et al. [18] where their results in a genetically-based mouse model of AD are very impressive.

## 5. Conclusions

The results of simulation studies show that inducing gamma oscillations in the hippocampus may reduce Alzheimer’s disease related pathology. Pathologically higher transfer entropy values after gamma induction returned to values comparable to the control model.
